# Holocene global mean surface temperature, a multi-method reconstruction approach

**DOI:** 10.1038/s41597-020-0530-7

**Published:** 2020-06-30

**Authors:** Darrell Kaufman, Nicholas McKay, Cody Routson, Michael Erb, Christoph Dätwyler, Philipp S. Sommer, Oliver Heiri, Basil Davis

**Affiliations:** 1 Northern Arizona University, School of Earth and Sustainability, Flagstaff, AZ 86011 USA; 2 University of Bern, Institute of Geography and Oeschger Centre for Climate Change Research, Bern, 3012 Switzerland; 3 University of Lausanne, Institute of Earth Surface Dynamics, Lausanne, 1015 Switzerland; 4 Institute of Coastal Research, Helmholtz-Zentrum Geesthacht - Centre for Materials and Coastal Research, Max-Planck-Straße 1, Geesthacht, 21502 Germany; 5 University of Basel, Department of Environmental Sciences, Basel, 4056 Switzerland

**Keywords:** Palaeoclimate, Climate and Earth system modelling

## Abstract

An extensive new multi-proxy database of paleo-temperature time series (Temperature 12k) enables a more robust analysis of global mean surface temperature (GMST) and associated uncertainties than was previously available. We applied five different statistical methods to reconstruct the GMST of the past 12,000 years (Holocene). Each method used different approaches to averaging the globally distributed time series and to characterizing various sources of uncertainty, including proxy temperature, chronology and methodological choices. The results were aggregated to generate a multi-method ensemble of plausible GMST and latitudinal-zone temperature reconstructions with a realistic range of uncertainties. The warmest 200-year-long interval took place around 6500 years ago when GMST was 0.7 °C (0.3, 1.8) warmer than the 19^th^ Century (median, 5^th^, 95^th^ percentiles). Following the Holocene global thermal maximum, GMST cooled at an average rate −0.08 °C per 1000 years (−0.24, −0.05). The multi-method ensembles and the code used to generate them highlight the utility of the Temperature 12k database, and they are now available for future use by studies aimed at understanding Holocene evolution of the Earth system.

## Introduction

During the two millennia prior to the 20^th^ Century, global mean surface temperature (GMST) cooled at a rate of roughly −0.15 °C per 1000 years^1^. Not well known, however, is: when did the multi-millennial cooling begin, and has recent global warming exceeded the maximum GMST of the Holocene? The only previous GMST reconstruction for the Holocene based on multi-proxy data^2^ showed maximum warmth around 7000 ± 2000 years ago (7 ± 2 ka BP, where ‘BP’ is relative to 1950) followed by multi-millennial global cooling. This cooling trend occurred while the atmospheric concentrations of greenhouse gases were increasing. Liu *et al*. (ref. ^3^) coined the term “Holocene temperature conundrum” to highlight the contradiction between the cooling indicated by proxy evidence versus the warming simulated by global climate models, a trend reinforced in the most recent generation of climate models^4^.

A more extensive database of paleo temperature time series is now available^5^, enabling a more robust reconstruction of the evolution of Holocene GMST and associated uncertainties than was available previously. More accurate constraints on the timing and magnitude of GMST are important for understanding how energy imbalances (climate forcings) are enhanced or diminished by feedbacks in the Earth system. The GMST reconstruction is also needed to place recent global climate change into the longer-term context of natural climate variability.

The Holocene temperature reconstructions generated in this study are the basis of the current paper, which is an ‘Analysis’ article type used by *Scientific Data* to highlight data reuse, including the statistical methods and supporting source code used to derive the conclusions. This Analysis complements the Temperature 12k data descriptor^5^, which explains the methods used to assemble the database and summarizes the major features of the underlying records. The database is the most comprehensive global compilation of previously published Holocene proxy temperature time series currently available. It comprises a quality-controlled collection of high-resolution time series (average sample spacing of 164 years) with well-established time scales (average of 1.0 age control points per 1000 years) that was selected from a much larger collection of temperature-sensitive proxy records. The multi-proxy database includes a total of 1319 paleo-temperature records from 470 terrestrial and 209 marine sites where ecological, geochemical and biophysical proxy indicators have been used to infer past temperature changes. Among the variety of proxy types, alkenones and isotopes are the dominant sea-surface temperature proxies, whereas pollen and chironomids are the most common terrestrial temperature proxy types. Most of the records (97%) are available as quantitative temperature reconstructions calibrated to °C, whereas the remaining 42 records represent non-quantitative temperature-sensitive proxy records.

There is no currently accepted best approach to reconstructing GMST based on multi-proxy data. Multiple statistical procedures have been developed to generate time series of paleoclimate variables over large regions and to quantify their uncertainties. Because each one is based on different assumptions and procedures, they can result in different reconstructions (e.g., refs. ^1,6^). Here, we apply five different statistical methods to the Temperature 12k database to reconstruct global and latitudinal temperatures over the past 12,000 years. The analysis quantifies the extent to which the overall result depends on the choice of reconstruction procedures. The resulting multi-method ensemble of plausible temperature histories captures the integrated uncertainties associated with multiple sources of errors and methodological choices.

## Results

### Global mean surface temperature reconstructions

#### Composites

The five reconstruction methods used in this analysis are all variations on compositing (aka, ‘stacking’) aimed at quantifying the average temporal patterns in the underlying proxy data. The major features of each method are listed in Table 1. They result in time series (index) rather than spatially resolved field reconstructions. Two of the methods — composite plus scale (CPS) and pairwise comparison (PAI) — generate composites by standardizing the temperature variance across proxy time series, then restoring it to a target value at the aggregated level. The term “scaling” is used in this paper to refer to matching the variance of a composite to that of a target, a technique commonly used for large-scale climate reconstructions that rely on proxy data that have not been calibrated to temperature, including those focusing on the past millenium^1,6,7^. In contrast, three of the methods — standard calibrated composite (SCC), dynamic calibrated composite (DCC), and generalized additive model (GAM) — generate composites using the native variance of the calibrated proxy data, without scaling. These methods apply to the 97% of the proxy records in the database that are presented in units of °C. Each of the methods requires many choices involving the specific procedures and formulas that are used to generate the reconstructions and their associated uncertainties. Generally, when there was no clear justification otherwise, we chose different alternative procedures for analogous steps among methods, with the goal of expanding the range of plausible outcomes.

**Table 1:** Major features of the five reconstruction methods and their uncertainty estimates.

	SCC	DCC	GAM	CPS	PAI
***Method includes:***					
Binning	100 yr	100 yr	None	100 yr	100 yr
Time series aligning	Mean temperature of 5–3 ka subtracted from each data point	Mean of each record iteratively adjusted to minimize differences among records within a latitude zone	Mean temperature of 5–3 ka subtracted from each data point	Mean of random 3000-year-long period (<7 ka) iteratively adjusted to minimize differences within a latitude zone	Not applicable
Variance standardizing	Not applicable	Not applicable	Not applicable	±1 SD over random 3000-year period	Rank-based normalization
Target variance scaling	Not applicable	Not applicable	Not applicable	2k reconstructions based on the same CPS procedure	2k temperature field reconstruction
Uncalibrated proxies	No	No	No	Yes	Yes
Total records	761	782	761	824	824
Local gridding	Yes	No	Yes	No	No
30° zonal bands	Yes	Yes	Yes	Yes	Yes
Ensemble members	500	500	500	500	500
***Uncertainties include:***					
Temperature calibration (Table 2)	Normal distribution	Auto-correlation model	Normal distribution	Auto-correlation model	Auto-correlation model
Chronology	±5%	BAM	GIBBS	BAM	BAM
Time-series alignment window	No (constant)	Yes (variable)	No (constant)	Yes (variable)	No (constant)
Target variance	Not applicable	Not applicable	Not applicable	CPS-based 1000-year reconstruction	Field reconstruction over 1000 yr
***Mid-Holocene (6.5–5.5 ka) global mean temperature relative to 1800–1900:***					
Median (°C)	0.50	0.50	0.44	1.08	0.42
5^th^, 95^th^ percentiles	0.20, 0.84	0.19, 0.79	0.10, 0.77	0.40, 1.84	0.22, 0.72

SCC: Standard Calibrated Composite; DCC: Dynamic Calibrated Composite; GAM: General Additive Model; CPS: Composite Plus Scale; PAI: Pairwise Comparison.

Among the procedures applied consistently across all five methods was the area-weighting of latitude-zone composites to generate the global composite of proxy records. Specifically, the GMST was calculated as the mean of the six 30° latitude averages, each weighted by the proportion of Earth’s surface area represented by that band (0.067, 0.183, and 0.25 for the high, middle, and low latitude bands, respectively, assuming a spherical Earth). This approach reduces the spatial bias of sample sites in the Northern Hemisphere while providing insights into the Holocene evolution of the latitudinal temperature gradient (cf. ref. ^8^).

#### Uncertainties

For each method, a 500-member ensemble of plausible reconstructions was generated to facilitate a probabilistic analysis of uncertainties. Ensembles were generated for all methods by sampling uncertainties related to chronology and proxy-inferred temperatures for the individual records. In addition, ensembles for some methods reflect different choices for selecting the time window over which to align temperatures. The two methods that rely on variance scaling also incorporate uncertainties in the reconstruction target. For the proxy temperature errors, we followed previous paleoclimate syntheses aimed at large-scale reconstructions (e.g., ref. ^2^) by applying a single uncertainty estimate to each proxy type. These global proxy uncertainties (Table 2) are based on values reported in the literature, along with the output of Bayesian-formulated calibrations for four of the marine proxy types (Supplemental Table 1).

**Table 2:** Uncertainties used for proxy-based temperatures in this study. The individual studies used to derive these values are in Supplemental Table 1.

Archive Type	Proxy Type	Temperature uncertainty (°C)		
		Summer	Winter	Annual
marine sediment	alkenone			1.7
marine sediment	δ^18^O			2.1
marine sediment	Mg/Ca	1.9	1.9	1.9
multiple archives	TEX_86_			2.3
marine sediment	foraminifera	1.3	1.4	1.3
marine sediment	dinocyst	1.7	1.2	1.2
multiple archives	diatom	1.1		
marine sediment	radiolaria	1.2		
multiple archives	pollen	2.0	3.0	2.1
multiple archives	GDGT^a^			2.9
multiple archives	stable isotopes			default
lake sediment	various^b^			default
lake sediment	chironomid	1.4		
glacier ice	various^c^			default
midden	macrofossils			default
wood	tree ring width			default

^a^MBT'5Me, MBT’'-CBT, MBT-CBT, MBT/CBT, Branched GDGT Fractional Abundance.

^b^BSi, TOC, chlorophyll, particle size, Mg/Ca, diatom, alkenone.

^c^melt-layer frequency, borehole, gas, isotope diffusion, bubble frequency.

#### Reference period

The mean temperature of the 1800–1900 bin of each composite was used as the pre-industrial reference period, that is, the mean 19^th^ Century temperature was set to anomaly of 0 °C. In practice, the mean temperature of the entire record was first removed from each ensemble member, which avoids the issue of different reconstructions using different internal reference periods. Then the ensemble median at 1800–1900 was subtracted for each method separately, which avoids the issue of some individual records not including data within the 1800–1900 bin. The mean temperature of the 19^th^ Century, in turn, is essentially equivalent to the reference for pre-industrial times as stipulated by the Intergovernmental Panel on Climate Change (IPCC), namely 1850–1900. On the basis of the PAGES 2k multi-method ensemble median reconstruction^1^, the difference between the GMST of our reference century and the IPCC’s half century is –0.03 °C, essentially negligible for our purposes.

### Similarities and differences among reconstructions

**Fig. 1:**
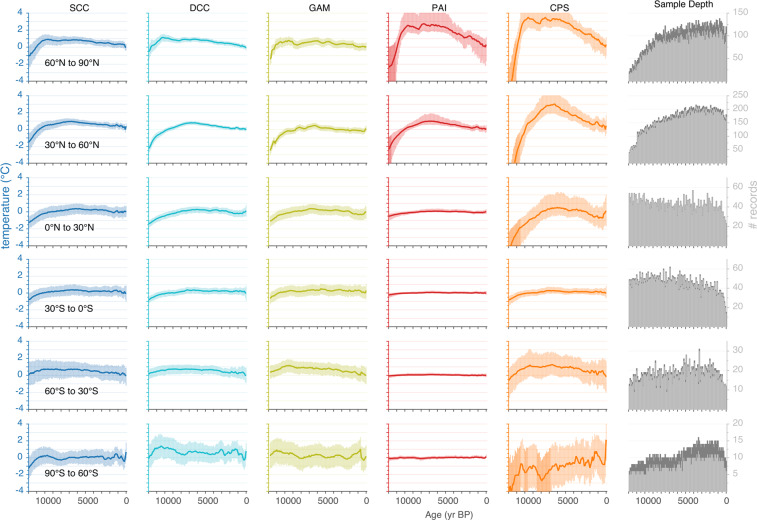
Reconstructed mean annual temperature for each of the five methods (columns) and six 30° latitude bands (rows). Colored lines are ensemble medians. The uncertainties for each method take into account different sources of errors as described in Methods and listed in Table 1. The methods include Standard Calibrated Composite (SCC), Dynamic Calibrated Composite (DCC), Composite Plus Scale (CPS), Pairwise Comparison (PAI) and Generalized Additive Model (GAM). Temperature anomalies are relative to 1800–1900. The number of proxy records represented within each 100-year time step is shown in the sixth column (sample depth). Light-grey vertical bars are the number of records calibrated to temperature and the dark-grey bars are the number of non-calibrated proxy records. The actual number of records used differs slightly among the reconstruction methods depending on limitations of each.

In Fig. 1, the median ensemble member for each of the five reconstruction methods is shown (columns) with uncertainty bands representing 90% of the ensemble members for each of the six latitudinal zone composites (rows). At the multi-millennial to millennial scale, the different methods all yielded similar overall shapes according to latitude, including the relative magnitude of warming during the first two millennia, the timing of peak warmth, and the relative magnitude of the multi-millennial cooling trend that followed. At the multi-centennial scale, the reconstructions from the different methods show similarities as well. At 60–90°N, for example, the initial peak temperature at around 10 ka is followed by a reversal around 8.5 ka, which is exhibited in all but the smoother SCC reconstruction; this is succeeded by a second temperature maximum around 7 ka. The major differences among the methods is the greater range of temperatures both within and among the latitudinal zones that are reconstructed by PAI and CPS, the two methods that rely on variance scaling. These two methods also generated the most contrasting uncertainty bands, which reflects the different procedures used to calculate them (Methods). The reconstruction methods differ slightly in the number of records that are represented at each time step (Fig. 1), which also reflects the different procedures and associated limitations (Methods).

**Fig. 2:**
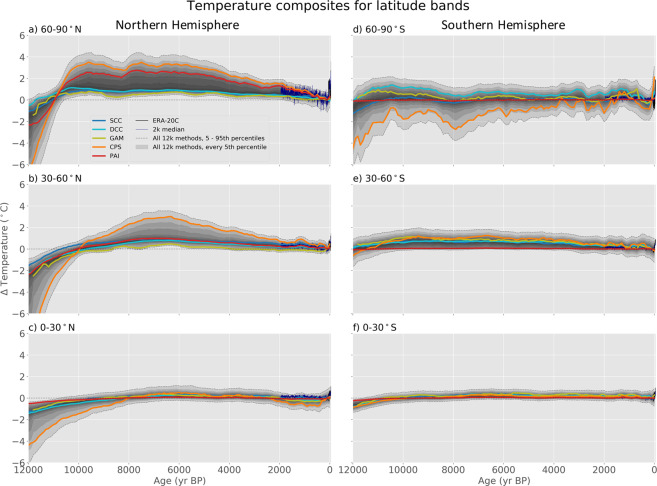
Reconstructed mean annual temperatures from the Temperature 12k database using different reconstruction methods for each of the six 30° latitude bands. Colored lines are the ensemble medians of each of the five reconstruction methods (abbreviations defined in Fig. 1 caption). Gray shading represents every 5^th^ percentile of the 2500 ensemble members from all methods; the 5^th^ and 95^th^ percentiles are indicated by dotted lines. The fine blue line is the median latitude-band 2000-year, multi-model temperature field reconstruction from Neukom *et al*. (ref. ^9^), which was based on data from PAGES 2k Consortium (ref. ^10^). Latitude-band temperatures from ERA-20C (ref. ^26^) (black) are also shown. Temperature anomalies are relative to 1800–1900.

In Fig. 2, the median of the ensembles for each method is shown along with the distribution of the combined, multi-method, 2500-member ensemble for each of the six latitudinal zones. This figure further illustrates the similarities and differences among the outcomes of the reconstruction methods as described above. It also displays the annually resolved temperatures over the past 2000 years from the multi-method temperature-field reconstruction of Neukom *et al*. (ref. ^9^), which was based on the PAGES 2k temperature database^10^.

### Consensus global temperature reconstruction

**Fig. 3:**
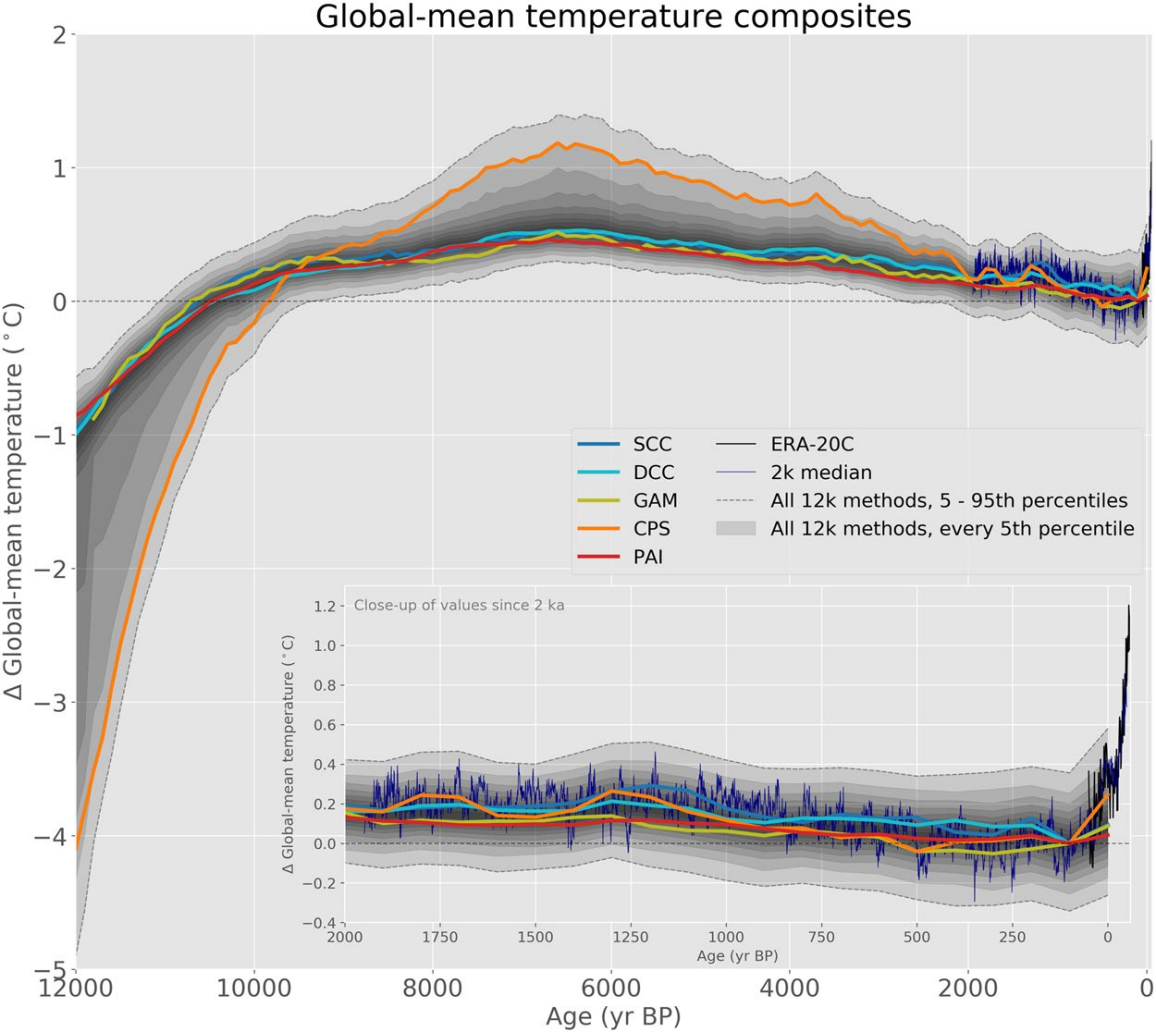
Global mean surface temperature from the Temperature 12k database using different reconstruction methods. The fine black line is instrumental data for 1900–2010 from the ERA-20C reanalysis product^26^. The inset displays an enlarged view of the past 2000 years. See Fig. 2 for additional explanation.

In Fig. 3, the median of the ensembles for the GMST reconstruction from each of the five methods is shown along with the combined distribution of the 2500 ensemble members. Because we do not have an objective means to determine which of the five reconstruction methods is most accurate, we combine the ensemble members from all methods to generate this consensus GMST reconstruction, the same approach used by PAGES 2k Consortium (ref. ^1^) and Neukom *et al*. (ref. ^9^) in their 2000-year GMST reconstructions. This 2500-member, multi-method ensemble incorporates uncertainties and differences that arise from different reconstruction procedures and choices. We recommend that future users of this reconstruction use the full ensemble when considering the plausible evolution of Holocene GMST. When representing the multi-method reconstruction as a single time series, the median of the ensemble may be the best, along with the 90% range of the ensemble to represent the uncertainty.

### Timing and magnitude of peak Holocene global temperature

**Fig. 4:**
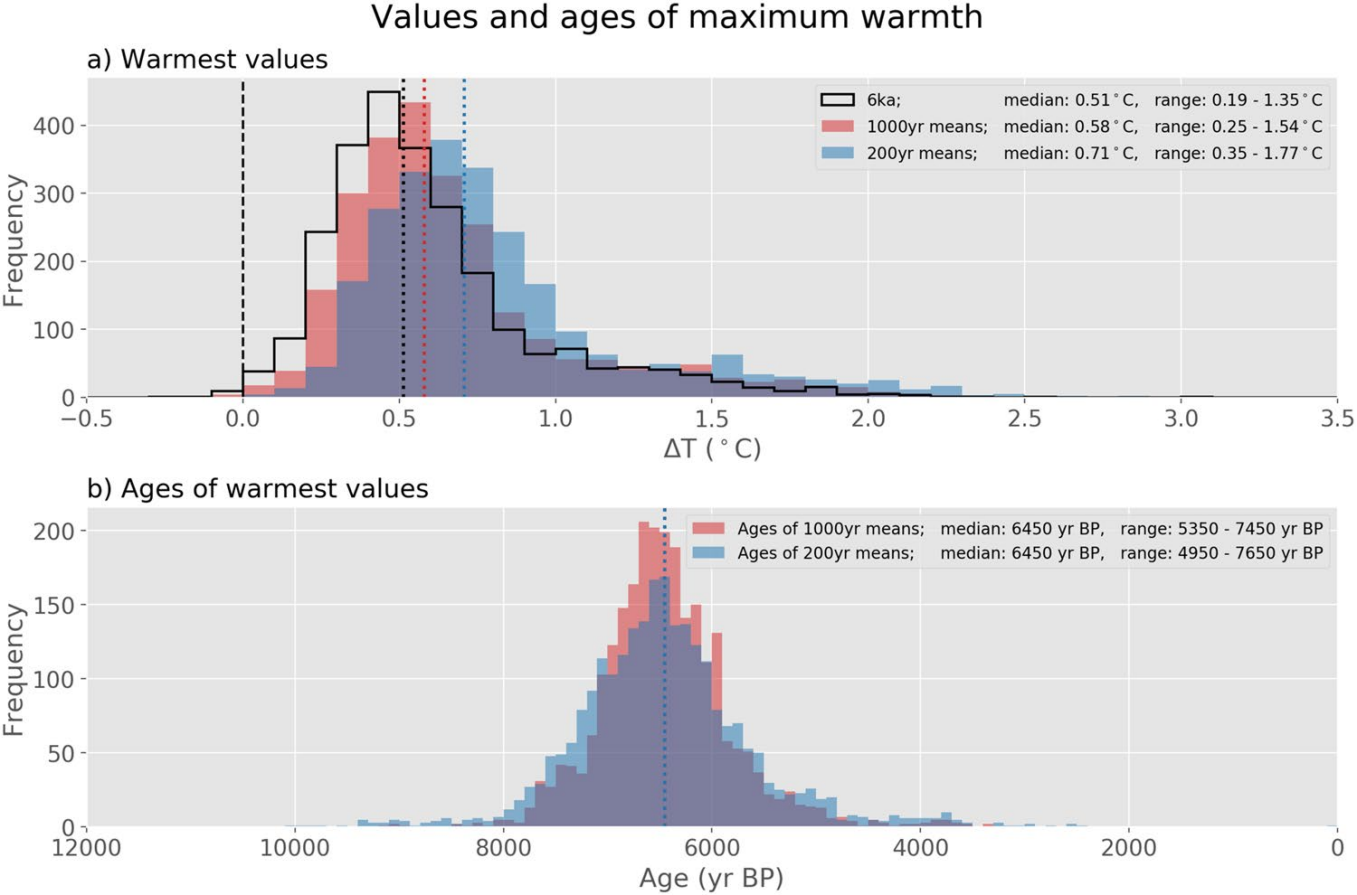
Magnitude and timing of peak temperatures from all 2500 members of the multi-model ensemble. (**a**) Warmest 1000-year (red) and 200-year-long (blue) intervals of the Holocene (colors), along with the temperature of the 1100-year period centered on 6 ka (black outline). Temperature relative to 1800–1900 reference period. (**b**) Timing of warmest 1000-year (red) and 200-year-long (blue) intervals. Values represent the mid-point of the time window. Dotted vertical lines are medians. Medians and 90^th^ percentile ranges are listed in the legends.

The combined 2500-member, multi-method ensemble was analyzed to determine the timing and magnitude of the peak GMST. To bracket the likely range of the temporal resolution of the GMST reconstruction, we focus on intervals of 1000 and 200 years, and quantify the difference in their magnitude and timing of peak warmth (Fig. 4). The distribution of ensemble members shows that, on average, the warmest millennium of the Holocene was centered on 6.5 ka and was 0.6 °C (0.3, 1.5) warmer than the 1800–1900 reference period (based on the median of the individual ensemble members, with 5^th^ and 95^th^ percentiles). The warmest 200-year-long interval was also centered on 6.5 ka and was 0.7 °C (0.3, 1.8) warmer than the 19^th^ Century. Therefore, there is little difference in the timing and magnitude of peak warmth when evaluated for either the centennial or the millennial time scale.

The distribution of peak global temperatures during the Holocene can also be compared with recent temperatures. The GMST of the past decade (2011–2019) averaged 1 °C higher than 1850–1900^11^. For 80% of the ensemble members, no 200-year interval during the past 12,000 years exceeded the warmth of the most recent decade. For the other 20% of the cases, which are primarily from the CPS reconstruction, at least one 200-year interval exceeded the recent decade. This comparison is conservative in context of temperatures projected for the rest of this century and beyond, which are very likely to exceed 1 °C above pre-industrial temperature^12^. Such projections place the temperature of the last decade into a long-term context that is more comparable with the Holocene GMST reconstruction. Furthermore, if the reconstruction is influenced by a Northern Hemisphere summer bias (discussed below), then the peak warmth would be overestimated and the recent warming would therefore stand out even more in comparison.

Nonetheless, comparing average temperatures between intervals of different durations can be problematic because shorter intervals tend to capture more variability (including maximum warmth) than when time series are averaged over longer intervals. In addition, age inaccuracies that exceed the scale of the sample binning can lead to smoothing when records are averaged. And even well-dated proxy time series based on marine and lake sediments are often smoothed by biological and physical processes that disturb the sediment-water interface, thereby time-averaging the paleo-environmental signal, which can further reduce the variability represented by the proxy record^13^. The relatively minor warming during the 20^th^ Century in our reconstructions can probably be attributed to these processes and to the likelihood that samples within the 20^th^ Century bin are biased toward the early part of the century because recovering the very top of an aquatic sedimentary sequence with high water content can be challenging^14^.

### Cooling trend following peak warmth

**Fig. 5:**
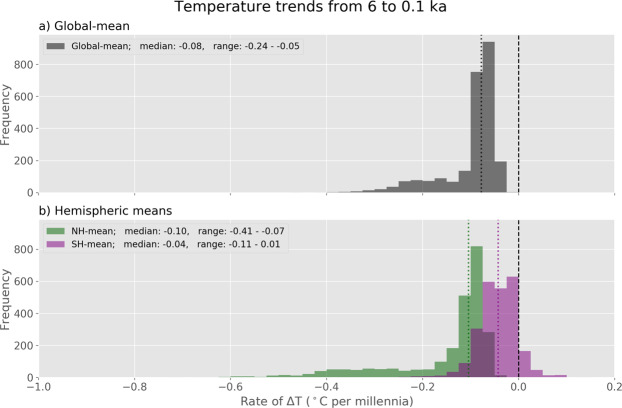
Temperature trends from 6.0 ka to 0.1 ka based on linear regression of multi-method ensemble members. (**a**) Global mean. (**b**) Hemispheric means based on area-weighted averages of three zonal bands for each hemisphere. The distribution of values for all 2500 ensemble members is shown, with median values marked as vertical lines. Medians and 90^th^ percentile ranges are listed in the legends.

The multi-method ensemble was further analyzed to determine the rate of cooling following the global Holocene thermal maximum. For this analysis we used the ensemble members from each of the six latitude zones so the trends in the two hemispheres could be treated separately. A least-squares linear regression was used to quantify the temperature trend between 6.0 and 0.1 ka in each ensemble member, where 0.1 ka is represented by the 1800–1900 time step. The results show (Fig. 5) that, for the Northern Hemisphere, 100% of the 2500 ensemble members cooled following 6 ka, with an area-weighted average rate of −0.10 °C per 1000 years (−0.41, −0.07). For the Southern Hemisphere, 90% of the ensemble members cooled, with an area-weighted average rate of −0.04 °C per 1000 years (−0.11, 0.01). For the global reconstruction, 100% of the ensemble members show a cooling trend, with an average rate of −0.08 °C per 1000 years (−0.24, −0.05).

## Discussion

Among the five reconstruction methods, CPS stands out prominently with its large temperature changes (Fig. 3), especially in the Northern Hemisphere (Figs. 1 and 2). For example, the median ensemble member of the CPS reconstruction shows that GMST warmed by about 3.9 °C between 12 and 10 ka compared to about 1.1 °C for the other methods. The median GMST during the period centered on 6 ka, the long-standing mid-Holocene target for paleoclimate modeling experiments (e.g., ref. ^15^) was 1.1 °C warmer than the 19^th^ Century in the CPS reconstruction compared to about 0.4–0.5 °C for the other methods (Table 1).

**Fig. 6:**
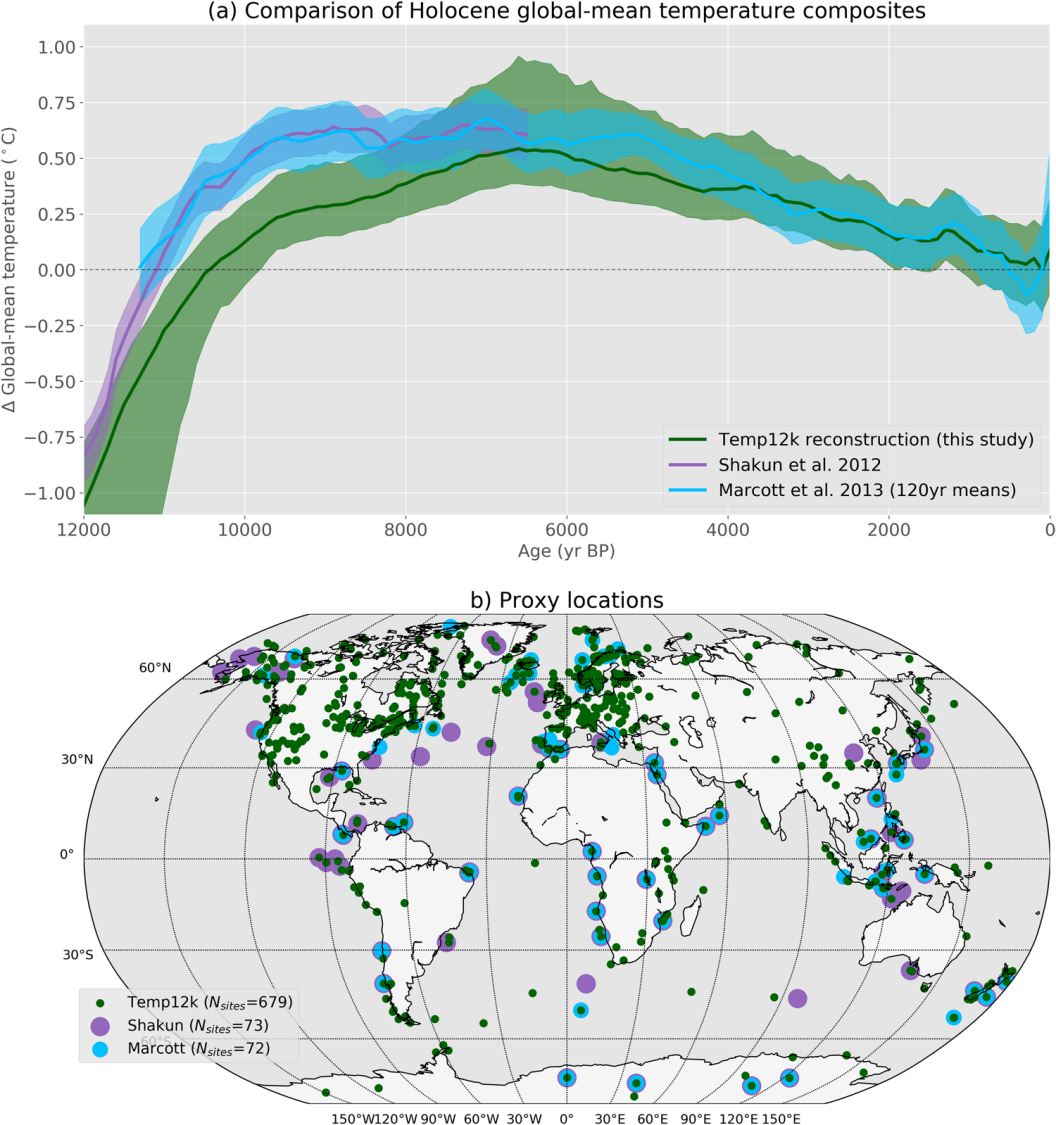
(**a**) Multi-method median global mean surface temperature reconstruction from this study compared with previous reconstructions, and (**b**) locations of proxy data sites. Uncertainty bands are ± 1 SD and 16–84% range of the Temperature 12k multi-method ensemble. The reconstruction of Marcott *et al*. (ref. ^2^) was binned into 120 year means, centered on the same years as the Temperature 12k reconstruction, and shifted to match our reference period of 1800–1900 (∆T = 0 °C). The reconstruction of Shakun *et al*. (ref. ^16^) for the early Holocene was aligned to that of Marcott *et al*.’s (ref. ^2^) over their period of overlap.

There are few published proxy-based reconstructions of Holocene GMST for comparison (Fig. 6). For the period of 12 to 10 ka, Shakun *et al*.’s (ref. ^16^) multi-proxy GMST reconstruction of the last deglacial transition shows a warming of about 1.3 °C. Similarly, Snyder’s (ref. ^17^) 2 Myr global temperature transformation of the marine oxygen-isotope record also indicates a warming of about 1.3 °C over this period. Both of these studies suggest that the early Holocene warming is exaggerated in the CPS reconstruction, by about a factor of three. For the mid Holocene (6.5–5.5 ka), Marcott *et al*.’s (ref. ^2^) reconstruction shows a GMST approximately 0.6 °C warmer than the 19^th^ Century (Fig. 6), and another proxy-data compilation focused on the mid-Holocene (6 ka) shows average land and sea-surface temperatures that are essentially indistinguishable from pre-industrial (Fig. 1c in ref. ^15^). Both of these studies suggest that the mid Holocene warmth is exaggerated in the CPS reconstruction. The recent pollen-based Holocene temperature reconstruction for North America and Europe^18^ also implies less mid-Holocene warming, although comparing global with regional reconstructions can be problematic.

Although it is an outlier, we do not have irrefutable evidence to exclude the CPS reconstruction, and cannot rule out the possibility that the other reconstruction methods underestimate the overall variance. The outcome of the CPS method depends on the validity of the target used for scaling, which is difficult to verify. The high amplitude of temperature changes reconstructed by CPS might reflect chronological and other uncertainties that average out century-scale temperature variance during the compositing, thereby increasing the relative magnitude of millennial-scale variance in the composite. When the composite is then scaled to the reconstructions of the past two millennia, which have more realistic century-scale variance, the millennial-scale variance (and thus the long-term trends) are artificially inflated. Nonetheless, as an independent approach, CPS contributes to a more complete sampling of the uncertainty space. We therefore retain CPS as one-fifth of the multi-method ensemble, and we focus on the median rather than the mean as the best representation of the ensemble central tendency. Excluding CPS from the ensemble does little to influence the median GMST reconstruction. For example, the mid-Holocene (6.5–5.5 ka) ensemble median is only 0.05 °C cooler when excluding the CPS members; namely, the five-method median is 0.51 °C (0.19, 1.35) versus 0.46 °C (0.17, 0.79) when excluding CPS members.

Reconstructing past temperature from proxy data relies on important assumptions that differ among proxy types and the methods used to convert proxy values to past temperatures, and can lead to biased or spurious results when violated^19–21^. The general similarity between our GMST reconstruction and boreal summer insolation might reflect a bias that can arise when proxy types that are sensitive to summer conditions are scaled to represent mean annual temperatures. This effect has been suggested for previous mean annual temperature estimates based on pollen^22^ and br-GDGTs^23^ among other proxy types, and was considered a possible explanation for the Holocene temperature conundrum^3^. The extent to which our GMST reconstruction is summer biased is difficult to ascertain, but the available evidence suggests that any such bias is limited. First, although the reconstructions in this analysis are based on site-level records that represent both annual and seasonal (summer or winter) temperatures, figure 8 of the Temperature 12k data descriptor^5^ shows that the global z-score composite that combines annual and seasonal records (*n* = 813) is indistinguishable from the composite based on annual records only (*n* = 612). Second, if the proxies are summer biased and if insolation was the dominant control on temperature, as simulated by climate models (e.g., ref. ^24^), then we would expect a long-term Holocene warming trend in the Southern Hemisphere, especially in the subtropics, which is not supported by our temperature reconstructions. Finally, a summer bias would be expected to influence different proxy types differently, yet z-score composites for each of the major proxy types in the Temperature 12k database show a similar shape (Fig. 4 in ref. ^5^). Similarly, we tested the influence of individual major proxy types by successively leaving one out of the GMST reconstructions and found that the overall pattern is not dependent on any one type. Moreover, temperature estimates based on pollen (the most abundant proxy type in the Temperature 12k database), which generally rely on conventional calibration methods keyed to modern training sets, are supported by temperature estimates from inverse modelling, an independent method that relies on a vegetation model to infer climate variables from a given vegetation community^25^.

Another potential issue in reconstructing GMST is the possible spatial bias of the sampling network. In this paper, the temperature histories of six 30°latitude bands were reconstructed separately and then averaged together with area weighting to mitigate the predominance of Northern Hemisphere proxies in the network. While this approach places increased emphasis on the relatively sparse Southern Hemisphere records, it keeps the Southern Hemisphere signal from being swamped by the more abundant Northern Hemisphere records. When processed this way, the general distribution of the Temperature 12k proxy network has been shown to accurately represent the GMST, as well as the average temperature of each of the six latitude bands (figures 9 and 10 in ref. ^5^).

The evolution of Holocene temperature reconstructed by the five methods are generally similar, although the amplitude of change is higher for CPS. The multi-method median reconstruction is very similar to the only other available multi-proxy, Holocene GMST reconstruction^2^ (Fig. 6). Our reconstruction, which is based on much more proxy data and multiple statistical methods, reinforces the mismatch between higher-than-pre-industrial GMST as represented by the proxy data versus the lower-than-pre-industrial GMST as simulated by climate models^3^. Understanding what caused this mismatch will undoubtedly be the focus of subsequent studies.

## Methods

### Input data

#### Holocene proxy temperature

The temperature reconstructions are based on v.1.0.0 of the Temperature 12k database^5^. For the majority of the 679 sites in the database, time-series data are available for either different proxy types or different seasons, or both. The analyses presented in this study are based on the subset of records that includes all proxy types, but only one seasonal record for each proxy type at a site. In other words, for proxy types with both annual and seasonal paleo-temperature time series, only the annual time series was used (‘*Season General*’ = ‘annual’ OR ‘summerOnly’ OR ‘winterOnly’). Included are 813 out of the 1339 records of which approximately half (612) represent mean annual temperature and the others represent either summer or winter temperature.

#### Two-thousand-year proxy temperature

The two reconstruction methods that rely on variance scaling (CPS and PAI) require a target for calibrating the normalized composite (in SD units) to temperature (in °C). We applied a different scaling procedure to the two reconstruction methods to highlight their differences. Unsurprisingly, the choice of scaling method has a large influence on the outcome of the reconstructions, and explains most of the difference between CPS and PAI, including the uncertainty bands. Both methods rely on a global database of proxy temperature records covering the past 2000 years (ref. ^10^) to bridge between the Holocene and the instrumental records. The variance in PAI is scaled to 100-year mean composites from the past 1000 years in the temperature field reconstruction calculated using multiple methods (ref. ^9^). For CPS, we emulated the same CPS methodology used for the past 12k for the six latitudinal bands in the PAGES 2k database, and then scaled those annually resolved 2k composites to zonal means from 1901–2000 calculated from the ERA 20C reanalysis^26^.

### Uncertainty estimates

#### Proxy-based temperatures

Quantifying uncertainties associated with proxy-derived paleo-temperatures is challenging^21^, and there are no standard procedures for their calculation or reporting. Some studies characterize uncertainties based on measurement errors, some report apparent calibration uncertainty estimates, while others report more rigorously cross-validated uncertainty values (e.g., based on leave-one-out cross-validation, bootstrapping, split sampling). For this reason, we follow previous paleo-climate syntheses aimed at large-scale reconstructions (e.g., ref. ^2^) by applying a single uncertainty estimate to each proxy type. We surveyed the literature to compile published errors, while striving for global coverage (Supplemental Table 1). Final error values used for individual proxy types (Table 2) are an estimate of the standard deviation, usually reported as the root mean square error of prediction, or the standard deviation of modern residuals relative to an inference method applied within a calibration dataset. They were adopted from previous large-scale calibration datasets and compilations of proxy-based temperature inferences, or they were calculated as averaged values of errors from multiple, usually regional calibration datasets (Supplemental Table 1). For marine records based on alkenones (U^K^’_37_), Mg/Ca, TEX_86_, and δ^18^O, we also included uncertainties represented by ensembles derived from resampling in a Bayesian framework (see ref. ^5^ for details). The values range from around ±1.2 °C for various marine microfossil assemblages (diatoms, radiolaria, dinocysts) to around ±3 °C for non-marine GDGTs and pollen-based winter temperatures. The median uncertainty across all proxies and seasons is ±1.7 °C. For proxy types not included in our literature review, we simply used the relatively conservative 75^th^ percentile of all values across the proxies and seasons (±2.1 °C). For the 42 non-calibrated proxy records (e.g., water isotopes), we used ±1 SD of the Holocene values as an assumed error on the standardized time series.

#### Chronology

Age control is a fundamental variable underlying paleoclimate time series. A comprehensive treatment of the age uncertainty would require recalculating the age model for each record using a uniform approach. While this has been done for many records in the Temperature 12k database, the process has not yet been completed for all records. Here, we apply three alternative approaches to accounting for the likely effect of age errors, as described for each of the methods below.

### Reconstruction method 1: Standard calibrated composite (SCC)

This procedure assumes that the temperature variance is accurate for all proxy time series. Calculations rely on values in °C rather than standardized SD units. The non-calibrated records in the Temperature 12k database were not used.

#### Aligning the time series

Many of the Holocene temperature records were originally published as temperature anomalies relative to different reference periods, rather than as absolute temperatures. To place all time series on a comparable scale, they were adjusted relative to the multi-millennial interval common to the most records, namely between 5 and 3 ka. Records that do not overlap with this interval (*n* = 21) were excluded. The time series were aligned by subtracting the mean temperature over this window from each data point within each time series. In other words, temperatures at this pre-compositing step were all expressed as relative to the 5–3 ka mean.

#### Compositing

The resulting time series were binned in 100-year intervals, and then gridded spatially using an equal-area grid (4000 total grid cells, each with area = 127,525 km^2^, following methods in ref. ^8^) to reduce the influence of clustered sites, especially in northern high latitudes. The binned time series within each grid cell were averaged. The gridded data were then averaged into six 30° latitudinal (zonal) bands.

#### Ensembling with uncertainties

An ensemble of time series was generated by sampling uncertainties in proxy values and chronology, which were added to each temperature data point for each record prior to binning and averaging. For temperature uncertainties, a random number was drawn from a normal distribution according to the proxy-specific values in Table 2. For age uncertainty, the age of each sample within a time series was multiplied by a random number drawn from a normal distribution with a mean of 1 and a standard deviation of 0.05 (i.e., ±5% age uncertainty). To preserve the stratigraphic order of the time series, the same random value was applied to all samples. The above process was repeated for 500 iterations to generate a probabilistic distribution for each zonal composite.

### Reconstruction method 2: Dynamic calibrated composite (DCC)

This procedure is similar to the SCC except, instead of using a single time window to align the records, this method applies a dynamic record-aligning procedure and it uses different approaches to characterizing the uncertainties.

#### Aligning the time series

The mean temperatures of each record were adjusted iteratively to optimally minimize the mean offset between each record and all other records within each latitudinal band over the past 12,000 years. This allowed time series with minimal or no overlap to be included in the composite, so all of the calibrated records in the database were used.

#### Compositing

No spatial gridding was applied, but the records were averaged within latitudinal bands.

#### Ensembling with uncertainties

For this method, errors in the proxy temperatures were either drawn from the posterior outputs of Bayesian temperature calibrations, where available in the database (*n* = 149, ref. ^5^), or simulated from the uncertainties summarized for each proxy type. For the simulation, the uncertainties were assumed to be auto-correlated, with an AR1 coefficient of 0.71, such that 50% of the variance in the uncertainties is auto-correlated. This model reflects the contribution of correlated bias, as well as uncorrelated uncertainties. Age uncertainties were simulated using the Banded Age Model (BAM)^27^, with a Poisson model and symmetric 5% over- and under-counting probabilities. Although BAM is designed for layer-counted age modelling, it produces reasonable first-order estimates of age uncertainty, and only requires the original ages as input. To demonstrate this, we compared the BAM-based ensembles produced here to 108 proxy datasets with age models produced by Bacon^28^. The root mean square error (RMSE) of the ensemble members was calculated relative to the median of each age ensemble over the past 12,000 years. The Bacon models had a median (mean) RMSE of 198 (216) years, whereas the BAM models produced here had a median (mean) RMSE of 251 (260) years. We find that BAM produces age ensembles with uncertainty ranges that are comparable to, but slightly larger than Bacon for these datasets. Although BAM does not accurately represent the full uncertainty structure, it ultimately produces similar, and slightly more conservative, results. Before compositing, DCC randomly selected separate temperature and age ensemble members, thereby effectively propagating these uncertainties through the subsequent compositing steps.

### Reconstruction method 3: Generalized additive model (GAM)

This method is closely related to SCC, but instead of computing the mean of the records within every 100-year interval, it fits a generalized additive model (GAM) through the ensemble. This model is then used to predict the temperature anomaly at a given time, as well as to generate the ensemble that reflects the uncertainties in the proxy records.

#### Ensembling with uncertainties

For proxy temperature uncertainties, we applied the proxy-specific values in Table 2 using a normal distribution. For chronological uncertainties, we assumed a standard deviation of 250 years at 12 ka, which decreased linearly to 50 years at 0 yr BP (1950). The ensemble was then generated using a GIBBS sampling algorithm^29^, a method commonly used for Bayesian inference (e.g., ref. ^30^). This algorithm is based on normal distributions for the individual samples (defined by the reported age and the associated age uncertainty) but each distribution is truncated such that the randomly sampled ages cannot be younger than the age of the previous (younger) sample nor older than the age of the consecutive (older) sample, thereby preserving the stratigraphic order of the time series.

#### Aligning and compositing the time series

All records within a grid cell were combined into a single time series by aligning the mean of the temperature ensemble with the overlapping time series ensemble of each of the records, starting with the longest time series. The combined records within a grid cell were adjusted to a mean of zero between 5 and 3 ka. Grid cells whose ensemble was based on fewer than 100 samples over this period were excluded. For each grid cell, a linear GAM with a penalized B spline^31^ was fitted through the ensemble of sample ages and temperatures. This model, which assumes a normal distribution, was then used to predict the expected value of the temperature anomaly for every 100 years, but only if there were more than 100 samples in the input ensemble within the 300-year interval around the target age. In addition, an ensemble of 500 members was generated as random samples from the posterior of the model. The grid boxes in each of the 30° latitude bands (for the expected values and for each of the sampled posteriors) were then averaged to generate the zonal reconstructions. These expected values are shown as solid lines in Figs. 1 and 2, and the spatially averaged samples from the posteriors have been used to derive uncertainty intervals.

### Reconstruction method 4: Composite plus scale (CPS)

This procedure is widely used for index reconstructions of climate variables (e.g., ref. ^1,7^). It recognizes that the temperature variance in proxy records, which invariably contain non-climatic noise, is significantly reduced when calibrated against instrumental time series and averaged to generate reconstructions (e.g., ref. ^32^). To help recover the lost variance, composites can be scaled to approximate that of a target with an accurate representation of variance.

#### Standardizing

Each time series was first standardized to have a mean of zero and a variance of ±1 SD (i.e., z-scores) over a random 3000-year-long interval younger than 7 ka. The mean of each time series was then iteratively adjusted to optimally minimize the mean offset between each standardized observation record and all of the other records within each latitudinal band.

#### Compositing

The resulting time series were then binned in 100-year intervals and then averaged, without geographic gridding, to generate each zonal composite.

#### Scaling

The mean and variance of each composite extending back to 12 ka was scaled to match that of a 1000-year-long target for the zone. The target was reconstructed by applying the same procedure described to the data from PAGES 2k Consortium^10^, with 50 ensemble members, and a reference period of 100 years during the past 2000 years for aligning the time series. The last millennium (1000–2000 CE) was used for scaling because the data density is very low in the first millennium for zonal bands south of 30° N latitude. The targets were themselves calibrated over the 20^th^ Century using the ERA-20C reanalysis product^26^ as an instrumental-based target. Each ensemble member in the 12k composite was scaled to a randomly selected target for the past 1000 years.

#### Ensembling with uncertainty

An ensemble of 500 composites was generated for each zone by repeating the above procedure while sampling across uncertainties in both chronology and temperature, following the same procedure as DCC, including a combination of Bayesian posteriors and the auto-correlation model for the temperature.

### Reconstruction method 5: Pairwise comparison (PAI)

This procedure recognizes that proxy data are not a perfect representation of temperature, and that random and systematic errors can lead to uncertainties in calibrating proxy data to temperature. PAI relaxes the assumption that the proxy data are linearly related to temperature and instead relies on the relative ranking of proxy values^33^.

#### Compositing

Each of the proxy time series was binned in 100-year intervals to simplify the computational demand by using evenly spaced temporal data, and to balance the influence of records, as PAI effectively weights each record as a function of the number of observations. The PAI code of Hanhijärvi *et al*. (ref. ^33^) was used to compare the order of successive data points in each proxy time series, regardless of the units or reference periods, and to calculate the relative agreement of this ordering for all proxy records. The strength of this agreement was used to reconstruct the relative changes in temperature through time. The regularization parameter value was set to 1 and optimized until the log likelihood changed by less than 10^−6%^.

#### Scaling

The mean and variance of each composite was scaled to match that of a 1000-year-long zonal target. The 2k target was extracted from the global temperature field reconstruction of Neukom *et al*. (ref. ^9^), which is based on the PAGES 2k Consortium (ref. ^10^) dataset and is represented by a multi-method ensemble.

#### Ensembling with uncertainty

An ensemble of 500 composites was generated for each zone by repeating the above procedure while sampling across uncertainties in both chronology and temperature, using the same procedure as DCC. To take into account scaling uncertainties, each 12k zonal composite was paired with, and scaled to, a different 2k zonal target randomly selected from the multi-method ensemble of temperature field reconstructions.

## Supplementary information

Supplementary Table 1

## Data Availability

The Temperature 12k database^5^ used to reconstruct the GMST is available in Linked Paleo Data format (LiPD) through the World Data Service (NOAA) Paleoclimatology^34^. The LiPD framework comprises JSON formatted, standardized files that are machine-readable in multiple programming languages for querying and data extraction. Proxy temperature records for the past 2000 years were compiled by the PAGES 2k Consortium^10^ and are available at: https://www.ncdc.noaa.gov/paleo/study/21171. The ERA-20C instrumental data reanalysis product is available at https://www.ecmwf.int/en/forecasts/datasets/reanalysis-datasets/era-20c. The primary outcomes for this study, including the ensemble temperature reconstructions for each method by latitude zone and globally are available as individual CSV files and merged as a netCDF file at figshare^35^ and at NOAA Palaeoclimatology^36^ (https://www.ncdc.noaa.gov/paleo/study/29712). A CSV file with the multi-method ensemble median and 5^th^ and 95^th^ percentiles for the latitudinal and global reconstructions is also available at both data repositories. Also available is the new ensemble of proxy time series generated for this paper by using BAM for age models and sampling uncertainties in the temperature domain, as described in Methods.

## Code availability

The code for working with the LiPD data files, including basic functions in three programming languages, is available on GitHub (https://github.com/nickmckay/LiPD-utilities). The code used to compute the temperature reconstructions, including the ensembles and the composites, and to the code for generating Figs. 2–6, is available at: https://github.com/nickmckay/Temperature12k under a MIT license^37^.
